# Supplementary material for: MetaQTL: a package of new computational methods for the meta-analysis of QTL mapping experiments
Source: BMC Bioinformatics. 2007 Feb 8;8:49. doi: 10.1186/1471-2105-8-49 (PMC1808479; doi:10.1186/1471-2105-8-49)
Supplement: Additional File 7 — MetaQTL Package : jar file and tutorial. This Zip archive contains both the MetaQTL JAR file and the files of the tutorial. [file 1471-2105-8-49-S7.zip › org.inra.metaqtl/doc/org/metaqtl/main/CmdLineParser.IllegalOptionValueException.html]

CmdLineParser.IllegalOptionValueException


|  |  |  |
| --- | --- | --- |
| **Overview** \| **Package** \| **Class** \| **Use** \| **Tree** \| **Deprecated** \| **Index** \| **Help** | |  |
| **PREV CLASS**   **NEXT CLASS** | **FRAMES**    **NO FRAMES**     **All Classes** |  |
| SUMMARY: NESTED \| FIELD \| CONSTR \| METHOD | DETAIL: FIELD \| CONSTR \| METHOD |  |


---


## org.metaqtl.main Class CmdLineParser.IllegalOptionValueException

```
java.lang.Object
  java.lang.Throwable
      java.lang.Exception
          org.metaqtl.main.CmdLineParser.OptionException
              org.metaqtl.main.CmdLineParser.IllegalOptionValueException
```

**All Implemented Interfaces:**: java.io.Serializable

**Enclosing class:**: CmdLineParser

---

``` public static class CmdLineParser.IllegalOptionValueException extends CmdLineParser.OptionException ```

Thrown when an illegal or missing value is given by the user for
an option that takes a value. `getMessage()` returns
an error string suitable for reporting the error to the user (in
English).

**See Also:**: Serialized Form

---

**Constructor Summary**

`CmdLineParser.IllegalOptionValueException(CmdLineParser.Option opt,
java.lang.String value)`


| **Method Summary** | |
| --- | --- |
| `CmdLineParser.Option` | `getOption()` |
| `java.lang.String` | `getValue()` |

**Methods inherited from class java.lang.Throwable**

`fillInStackTrace, getCause, getLocalizedMessage, getMessage, getStackTrace, initCause, printStackTrace, printStackTrace, printStackTrace, setStackTrace, toString`

**Methods inherited from class java.lang.Object**

`clone, equals, finalize, getClass, hashCode, notify, notifyAll, wait, wait, wait`

**Constructor Detail**

### CmdLineParser.IllegalOptionValueException

```
public CmdLineParser.IllegalOptionValueException(CmdLineParser.Option opt,
                                                 java.lang.String value)
```


**Method Detail**

### getOption

```
public CmdLineParser.Option getOption()
```

:   **Returns:**: the name of the option whose value was illegal (e.g. "-u")

---


### getValue

```
public java.lang.String getValue()
```

:   **Returns:**: the illegal value


---


|  |  |  |
| --- | --- | --- |
| **Overview** \| **Package** \| **Class** \| **Use** \| **Tree** \| **Deprecated** \| **Index** \| **Help** | |  |
| **PREV CLASS**   **NEXT CLASS** | **FRAMES**    **NO FRAMES**     **All Classes** |  |
| SUMMARY: NESTED \| FIELD \| CONSTR \| METHOD | DETAIL: FIELD \| CONSTR \| METHOD |  |


---
